# Impact of Hyperglycemia and Low Oxygen Tension on Adipose-Derived Stem Cells Compared with Dermal Fibroblasts and Keratinocytes: Importance for Wound Healing in Type 2 Diabetes

**DOI:** 10.1371/journal.pone.0168058

**Published:** 2016-12-19

**Authors:** Aurore Lafosse, Cécile Dufeys, Christophe Beauloye, Sandrine Horman, Denis Dufrane

**Affiliations:** 1 Université Catholique de Louvain, Brussels, Belgium; 2 Pole de Recherche Cardiovasculaire (CARD), Institut de Recherche Expérimentale et Clinique, Université Catholique de Louvain, Brussels, Belgium; 3 Novadip Biosciences, Mont-Saint-Guibert, Belgium; Universita degli Studi di Torino, ITALY

## Abstract

**Aim:**

Adipose-derived stem cells (ASC) are currently proposed for wound healing in those with type 2 diabetes mellitus (T2DM). Therefore, this study investigated the impact of diabetes on adipose tissue in relation to ASC isolation, proliferation, and growth factor release and the impact of hyperglycemia and low oxygen tension (found in diabetic wounds) on dermal fibroblasts, keratinocytes, and ASC *in vitro*.

**Methods:**

Different sequences of hypoxia and hyperglycemia were applied *in vitro* to ASC from nondiabetic (n = 8) or T2DM patients (n = 4) to study cell survival, proliferation, and growth factor release. Comparisons of dermal fibroblasts (n = 8) and keratinocytes (primary lineage) were made.

**Results:**

No significant difference of isolation and proliferation capacities was found in ASC from nondiabetic and diabetic humans. Hypoxia and hyperglycemia did not impact cell viability and proliferation. Keratinocyte Growth Factor release was significantly lower in diabetic ASC than in nondiabetic ASC group in each condition, while Vascular Endothelial Growth Factor release was not affected by the diabetic origin. Nondiabetic ASC exposition to hypoxia (0.1% oxygen) combined with hyperglycemia (25mM glucose), resulted in a significant increase in VEGF secretion (+64%, *p*<0.05) with no deleterious impact on KGF release in comparison to physiological conditions (5% oxygen and 5 mM glucose). Stromal cell-Derived Factor-1α (-93%, *p*<0.001) and KGF (-20%, *p*<0.05) secretion by DF decreased in these conditions.

**Conclusions:**

A better profile of growth factor secretion (regarding wound healing) was found *in vitro* for ASC in hyperglycemia coupled with hypoxia in comparison to dermal fibroblasts and keratinocytes. Interestingly, ASC from T2DM donors demonstrated cellular growth rates and survival (in hypoxia and hyperglycemic conditions) similar to those of healthy ASC (from normoglycemic donors); however, KGF secretion was significantly depleted in ASC obtained from T2DM patients. This study demonstrated the impact of diabetes on ASC for regenerative medicine and wound healing.

## Introduction

Type 2 diabetes is one of the three primary causes of impaired wound healing, which leads to chronic ulcer formation (15% to 25% of patients among approximately 380 million worldwide in 2013 and a prediction of 590 million in 2035) [1]. The pathophysiology is partially explained by impairment of angiogenesis, growth factor depletion (notably because of a lack of local expression of vascular endothelial growth factor [VEGF] and a nonfunctional HIF-1α signaling pathway), and dysfunction of diabetic endothelial progenitor cells and dermal fibroblasts (reduction of stromal cell-derived factor-1α [SDF-1α] secretion) [2–6]. Topical application of recombinant growth factors was proposed to promote wound healing of diabetic wounds but without drastic clinical efficiency, probably because of the short duration of the molecules at the site of wound [7].

Cell therapy is an attractive tool to restore the physiological context for diabetic wound healing because cells can contribute to tissue regeneration by effective and prolonged cytokine secretion at the wound site, immunomodulative properties, and cellular recruitments. Adipose-derived stromal cells (ASC) have been described since 2002 [8], are easily harvested by a subcutaneous biopsy, have high mesenchymal stem cell density per gram of adipose tissue, and possess differentiation, immunomodulative, and angiogenic properties [9] similar to those of bone marrow–derived stem cells. The ability of implanted ASC to differentiate into endothelial cells was described, as was their capacity to release large amounts of proangiogenic growth factors (particularly SDF-1α and VEGF) [10,11].

Several pre-clinical studies using diabetic rodent models demonstrated the positive effects of ASC implantation or injection to promote skin healing [12–15]. In contrast, alterations of ASC functions in diabetic rodent models were also described [6,16–18]. Because Desmet et al. demonstrated prolongation of hypoxia in diabetic mice *in vivo* [19], we postulated that hyperglycemia coupled with hypoxia may influence the function of ASC. Veriter et al. also demonstrated preliminary results concerning the capacity of VEGF released during hypoxia and normoxia conditions and its function in *in vitro* glucose concentrations. Significantly higher VEGF release was found for ASC at 25 mM in comparison to 5 mM glucose for different short time courses [9]. In addition, adipose tissue (the original source of ASC) remains very sensitive to chronic hyperglycemia (in type 2 diabetes), as evidenced by adipose tissue inflammation, which is characterized by infiltration of inflammatory cells, increased production of cytokines, and induced systemic insulin resistance [20,21]. They found that excessive calorie intake led to increased oxidative stress in the adipose tissue of mice with type 2 diabetes and promoted senescence-like changes, such as an increase of senescence-associated galactosydase activity, p53 expression, and production of pro-inflammatory cytokines.

Subsequently, this study investigated the growth factors release profile from non-diabetic and diabetic ASC in hypoxia and hyperglycemia, compared to DF and Kc. VEGF, SDF-1α and KGF were firstly selected because of their interesting properties in view to promote diabetic wound healing. VEGF and SDF-1α are angiogenic growth factors both involved in the promotion of wound healing by promotion of cell proliferation, migration and differentiation. VEGF is described as one of the most potent pro-angiogenic growth factor in the skin, by a direct effect on several cell types involved in wound repair (endothelial cells, macrophages, keratinocytes…) [22]. SDF-1α is mainly released by DF and is considered to play an important role in the trafficking of bone marrow-derived stem cells to the wound area and its promotion of wound repair and neovascularization [23]. The decrease in SDF-1α was also found to be responsible for decreased endothelial progenitor cells homing, and subsequent reduction of local angiogenesis and tissue repair [24]. KGF, secreted by various cell types (fibroblasts, endothelial cells,…) in the early stages of the wound healing process as well as during the later remodelling process), has been shown to induce migration and proliferation of Kc [25]. Other growth factors such as IGF-1, bFGF, and HGF were also described as major mitogenic effectors of Kc [26].

The aim of this study was first to evaluate *in vitro* the survival and function (proliferation and specific growth factor secretion) of human ASC from healthy patients in the in vitro diabetic wound environment (combination of hypoxia and hyperglycemia) compared to human dermal fibroblasts (DF) and keratinocytes (Kc). Also, the properties of ASC from human diabetic patients were assessed in the same conditions and compared to properties of nondiabetic ASC.

## Materials and Methods

This study was performed according to the guidelines of the Belgian Ministry of Health. All tissue procurement procedures were approved by the Ethical Committee of the Medical Faculty (Université Catholique de Louvain; national authorization number B40320108280). All patients signed consent to participate in the study after verbal and written information were provided by the principal investigator of the study. All consents were included and archived at the University Hospital Saint-Luc for each patient (for 30 years).

All materials were obtained from Lonza (Verviers, Switzerland), Sigma-Aldrich (St. Louis, MO, USA), or Invitrogen (Carlsbad, CA, USA) unless otherwise noted.

### I: In vitro impact of hyperglycemia and hypoxia on nondiabetic ASC, DF, and Kc

#### ASC and skin cell (DF and Kc) isolation and culture

Harvesting of adipose tissue (mean, 7.4 g) was performed by lipoaspiration using the Coleman technique in eight nondiabetic patients (Table 1) undergoing elective plastic surgery after informed consent and serologic screening [27]. Adipose tissue was digested with collagenase (in a water bath at 37°C for 60 minutes) to isolate ASC. Cells were finally suspended in a proliferation medium comprising Dulbecco's modified Eagle medium supplemented with 10% fetal bovine serum (FBS), L-glutamine (2 mM), and antibiotics (100 U/ml penicillin, 100 μg/ml streptomycin, and 1 μl/ml amphotericin B) [28].

**Table 1:** Non-diabetic ASC donor characteristics.

Donor	1	2	3	4	5	6	7	8
**Age (years)**	19	44	40	62	56	46	45	41
**Sex**	F	F	F	F	F	F	F	F
**Anatomical site**	Breast	Abdomen	Thorax	Back	Abdomen	Breast	Thorax	Thorax

Skin biopsy samples (mean, 1.5 cm^2^) were procured from the same nondiabetic patients (thoracic or abdominal region, n = 8). DF were isolated by explant technique from de-epidermized dermal biopsy specimens, cut in 2-mm × 2-mm fragments, and placed in plastic wells. A small volume of the proliferation medium was added to allow tissue adhesion to the plastic surface. After 24 hours of incubation at 37°C and 5% CO_2_, the proliferation media was replaced. This initial passage of the primary cells is referred to as passage 0. Dermal pieces were removed from the culture dishes when adherent cells were visible on the plastic surface surrounding tissue fragments. Cells were maintained in proliferation medium (replaced two times per week) up to passage 4 after sequential trypsinizations.

A commercial primary lineage of keratinocytes (n = 1; adult epidermal keratinocytes; ATCC, Manassas, VA, USA) was used to compare viability, proliferation, and secretion of these cells after stimulation in the same conditions as for ASC and DF (see below). Manufacturer instructions were followed for culture and trypsinization techniques.

At passage 4, ASC and DF were characterized for standard cell surface markers (anti-CD106, anti-CD105, anti-CD44, anti-CD45, anti-CD73, anti-CD90, anti-CD31, anti-CD11b, anti-HLA-DR, anti-Stro-1, anti-CD14, anti-CD34) by fluorescence-activated cell sorting (FACScan; BD Biosciences, San Jose, CA) as previously described. The differentiation capacity of ASC toward osteogenic lineage (Alizarin red and osteocalcin staining) was also tested at passage 4 [29,30].

#### Impact of oxygen tension and FBS on cell proliferation: EdU assay

The proliferation capacity of ASC, Kc, and DF was tested by direct DNA synthesis measurement via EdU (5-ethynyl-2’-deoxyuridine) incorporation (Invitrogen). Cells (ASC, DF, Kc) were cultured in 21.5cm^2^ dishes, at the density of 6000 cells/cm^2^. At passage 4, cell growth was stopped by 24hours of incubation without FBS, then stimulation was introduced for 48 hours, by addition of 1% FBS in presence of the different sequences of tested conditions (normo/hyperglycaemia and 0.1%/5%/21% oxygen tension). Positive control was determined by cells cultured in standard culture conditions (21% oxygen, 25mM glucose and 10% FBS), and cells cultured in the same conditions but without EdU in the medium were used as negative control. EdU incorporation was quantified after revelation by Alexa Fluor 488 dye, following supplier’s instructions. Labeled cells were quantified by FACScan [31].

#### Growth factor secretion profile

After trypsinization, cells (at passage 4) were seeded in 12-well culture plates in triplicate for testing in hypoxic chambers (Modular Incubator Chamber MIC-101; Billups-Rothenberg, Del Mar, CA, USA). Cells were incubated in proliferation media without FBS (to avoid interference in growth factor detection) and tested under different conditions: (i) hypoxia (0.1% O_2_ vs. 5% O_2_ at 5 mM glucose for a highly hypoxic environment vs. tissular oxygen tension, respectively); (ii) hyperglycemia (25 mM vs. 5 mM glucose at 5% O_2_ for hyper vs. normoglycemic environment, respectively); and (iii) a combination of both conditions (25 mM glucose plus 0.1% O_2_, reproducing a diabetic wound environment vs. 5 mM glucose plus 5% O_2_ for physiological conditions). After incubation for 24 hours in these conditions, cell culture supernatants were harvested individually and stored at −20°C for further growth factor (bFGF, IGF-1, HGF, VEGF, SDF-1α, and Keratinocyte growth factor [KGF]) quantification by enzyme-linked immunosorbent assay (Quantikine ELISA kit; R&D System, Minneapolis, MN, USA). Cellular viability was assessed immediately after hypoxic stress by 3-(4,5-dimethylthiazol-2-yl)-5-(3-carboxymethoxyphenyl)-2-(4-sulfophenyl)-2H-tetrazolium solution (MTS; Promega, Leiden, the Netherlands) assay. Hypoxic and glycemic stress tests and growth factor quantifications were performed in triplicate and duplicate, respectively. Results are expressed in picograms per millimeter.

### II: Impact of hyperglycemia and hypoxia on diabetic ASC

#### ASC isolation and function in type 2 diabetes patients

Adipose tissue biopsy samples were procured (see above) from four diabetic patients. ASC were isolated, cultured, characterized, and tested in the same *in vitro* experimental conditions as mentioned for nondiabetic ASC.

### Statistical analysis

Values are presented as means ± SD, except when otherwise specified. The one-sample Kolmogorov test and Q-Q plots were used to assess the normal distribution of values. Statistically significant differences between groups (with normal distribution) were tested by paired *t*-test and one-way analysis of variance with the Bonferroni post hoc test. Statistical tests were performed with PASW 18 (SPSS; IBM, New York, NY, USA); *p*<0.05 was considered significant.

## Results

After isolation and culture until passage 4, ASC were characterized by mesenchymal stromal cell surface marker profiles (CD44+, CD73+, CD90+, CD105+, CD45-, CD34-, CD14-, Stro-1, CD106-, CD31-, CD34-, HLA-DR-, and CD11b-) (Table 2), and osteogenic differentiation capacity was demonstrated by Alizarin red staining and osteocalcin immunohistochemistry.

**Table 2:** FACS characterization of ASC from non-diabetic and diabetic donors.

	Non-Diabetic	Diabetic
CD44	99.71	98.86
CD45	3.36	4.48
CD73	98.76	99.82
CD90	99.64	97.88
CD105	91.50	93.63

### I: Impact of oxygen tension and glucose concentration on ASC from healthy patients

#### Impact on cell growth and survival

Cell survival, proliferation, and growth factor secretion were evaluated: (i) in severe hypoxia (0.1% O_2_ tension vs. 5% O_2_ tension with fixed glucose level at 5 mM glucose); (ii) in hyperglycemia (25 mM vs. 5 mM glucose, with oxygen tension fixed at 5% O_2_); and (iii) in *in vitro* conditions mimicking the chronic diabetic wound environment (combination of hypoxia [0.1% O_2_] and hyperglycemia [25mM glucose] vs. physiological conditions at 5% O_2_ and 5 mM glucose).

**Fig 1:**
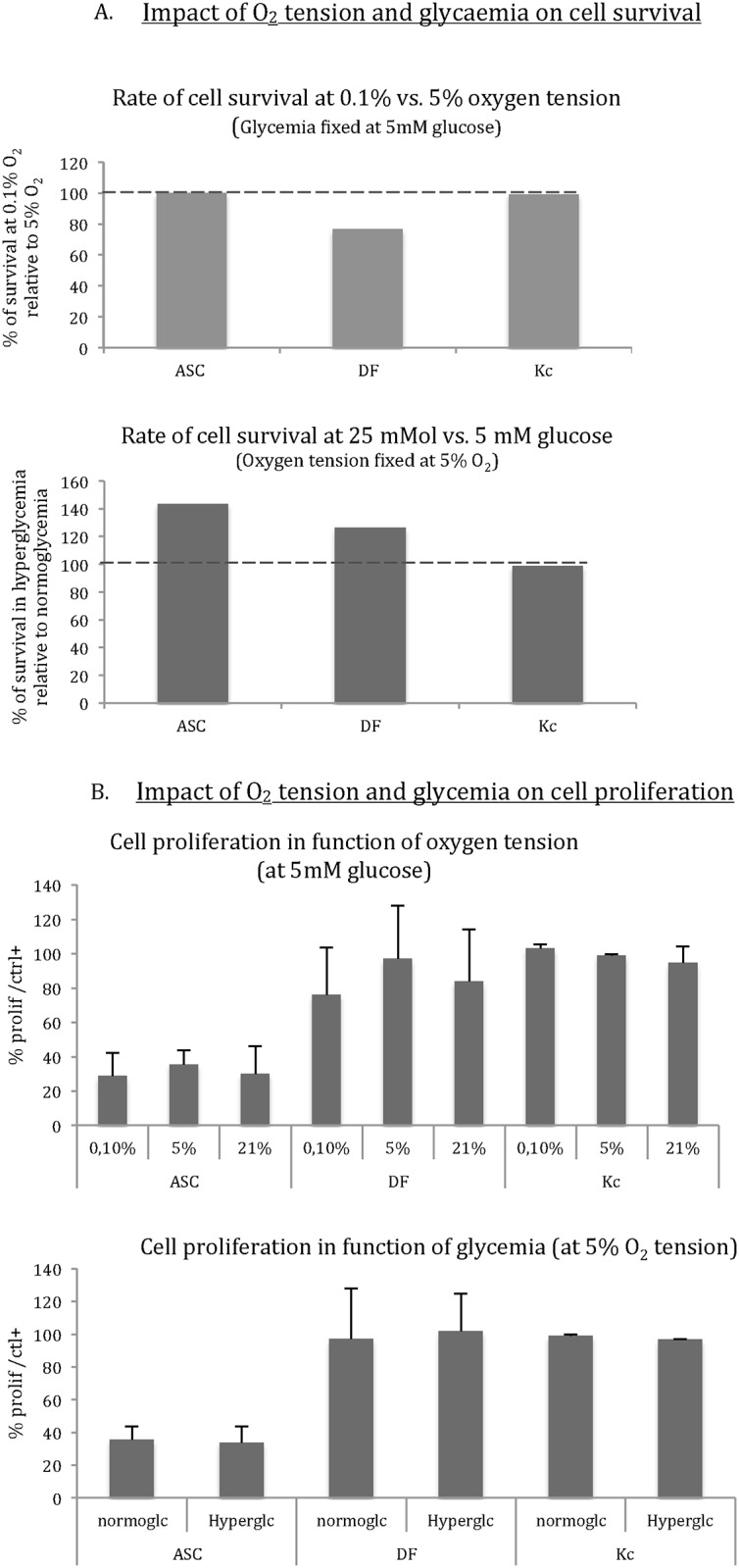
**A. Impact of hypoxia (in normoglycemia) and hyperglycemia (in normoxia) on cell survival**. Results are expressed as the percentage of the control (5% O_2_ and 5 mM glucose). After 24 hours in severe hypoxia (0.1% O_2_), survival rates of ASC and Kc were not affected, but the DF survival rate decreased to 76.8% of survival in 5%O_2_. Survival in 25 mM glucose was higher for ASC (143.7%) and DF (126.5%) in comparison to 5 mM glucose. **B. Impact of hypoxia and hyperglycemia on cell proliferation**. Oxygen tension (0.1%, 5%, or 21% O_2_) did not influence cell proliferation. Proliferation was also similar in normoglycemic and hyperglycemic conditions for each cell type. The lower general rate of ASC proliferation in all conditions demonstrated their sensitivity to lower FBS concentrations (1% in the stimulation culture media vs. 10% in standard conditions/control group).

Survival rates were not significantly modified after severe hypoxia for ASC (n = 8), DF (n = 8), and Kc (n = 1) (expressed as % of cell survival at 5% O_2_: 100%, 77%, and 99%, respectively, *p =* NS). Survival of ASC and DF was higher in 25 mM glucose, compared to normoglycemia (143.7% and 126.5%, respectively; Fig 1A).

The study of cell proliferation by EdU incorporation confirmed that the proliferation rate of each cell type was not influenced by oxygen tension (0.1% vs. 5% vs. 21% O_2_). Glycemia (25 mM vs. 5 mM) did not individually affect the proliferation rate of each cell type. Results were expressed as a rate of proliferation in comparison to the standard culture conditions (21% O_2_, 25 mM glucose, and 10% FBS) considered as a positive control (Fig 1B).

The lower ASC proliferation rate in comparison to DF and Kc in all conditions indicated greater sensibility of these cells to low FBS concentration in proliferation media (the proliferation rate was assessed under 1% FBS for 24 hours).

### Impact on growth factor release

**Fig 2:**
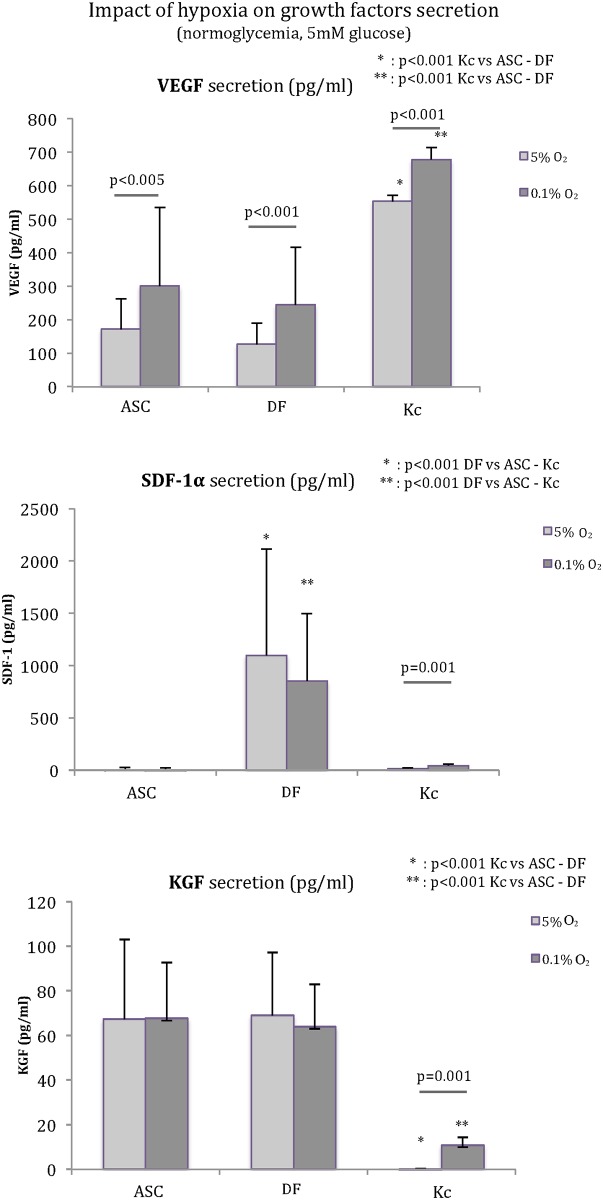
Impact of hypoxia on VEGF, SDF-1α, and KGF secretion. VEGF secretion was significantly stimulated by hypoxia (vs. tissular normoxia) for ASC (*p*<0.005), DF (*p*<0.001), and Kc (*p*<0.001). SDF-1α was mainly secreted by DF (*p*<0.001 compared to ASC and Kc) and was not significantly impacted by oxygen tension. ASC did not release SDF-1α, and secretion by Kc increased significantly in hypoxia (*p* = 0.001). KGF was mainly secreted by ASC and DF, but was modified only for Kc in hypoxia (*p* = 0.001).

The impact of hypoxia (0.1% O_2_) was studied for VEGF, SDF-1α, and KGF secretion (Fig 2). VEGF secretion was significantly increased in hypoxia for all cell types (ASC: +75%, *p*<0.005; DF: +92%, *p*<0.001; Kc: +22.5%, *p*<0.001) in comparison to 5% O_2_ tension. Significantly higher VEGF release was found for Kc versus ASC and DF at 0.1% and 5% O_2_ (*p*<0.001). Secretion of SDF-1 α was significantly higher for DF (*p*<0.001 in comparison with ASC and Kc) and was not significantly impacted by hypoxia (1098 ± 1017 pg/ml vs. 851 ± 645 pg/ml at 0.1% O_2_ vs. 5% O_2_, respectively). ASC did not secrete SDF-1α, whereas the small secretion from Kc was enhanced by hypoxia (42 ± 18 pg/ml vs. 14 ± 8 pg/ml at 0.1% O_2_ vs. 5% O_2_, respectively, *p* = 0.001). Significantly higher KGF release was found for ASC and DF compared to Kc with both oxygen tensions (*p*<0.001 for ASC and DF vs. Kc, *p* = not significant [NS] for ASC vs. DF). KGF secretion was only influenced by hypoxia in Kc (0 vs. 11 ± 4 pg/ml at 0.1% O_2_, *p* = 0.001).

**Fig 3:**
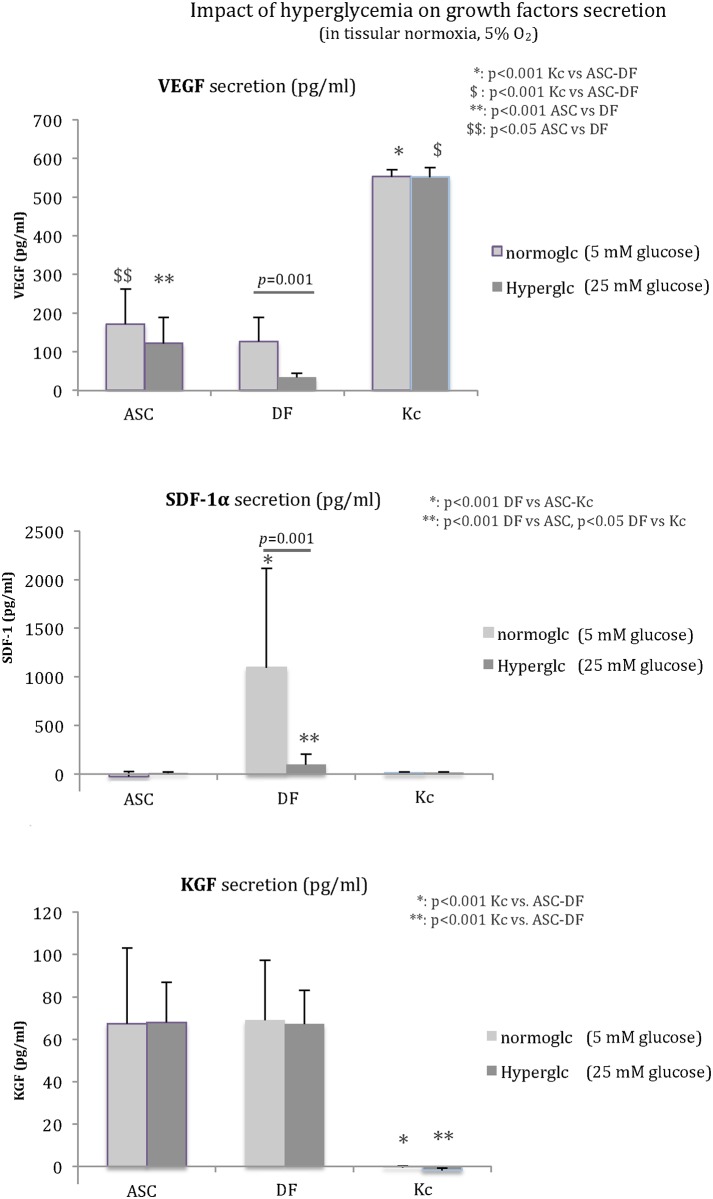
Impact of hyperglycemia on VEGF, SDF-1α, and KGF secretion. DF function was altered in hyperglycemia for VEGF and SDF-1α secretions (*p* = 0.001), whereas ASC and Kc were not affected. Kc produced higher rates of VEGF in each condition (*p*<0.001 compared to ASC and DF). VEGF secretion by ASC was higher than that by DF in both glucose concentrations (*p*<0.001 in normoglycemia and *p*<0.05 in hyperglycemia). KGF secretion was not influenced by glucose concentration for the three cell types. Kc release was significantly lower than that of ASC and DF (*p*<0.001).

In the same way, the impact of hyperglycemia (25 mM glucose vs. 5 mM glucose) was studied regarding VEGF, SDF-1α, and KGF secretions from ASC, DF, and Kc (Fig 3). VEGF secretion was not impacted by hyperglycemia for ASC and Kc, but was significantly reduced for DF (-29%, *p* = 0.001). Kc demonstrated the highest release of VEGF in both normoglycemia (553 ± 6 pg/ml vs. 172 ± 13 pg/ml and 127 ±62 pg/ml for Kc vs. ASC and DF, respectively, *p*<0.001) and hyperglycemia (552 ± 8 pg/ml vs. 122 ± 66 pg/ml and 35 ± 10 pg/ml for Kc vs. ASC and DF, respectively, *p*<0.001). In both normoglycemia and hyperglycemia, ASC produced significantly more VEGF than DF (+35.4% [*p*<0.05] and +253.3% [*p*<0.001], respectively).

SDF-1α was mainly secreted by DF in both 5mM glucose (1098 ± 1017 pg/ml vs. 0 pg/ml and 14 ± 8 pg/ml for DF vs. ASC and Kc, respectively, *p*<0.001) and 25mM glucose (101 ± 101 pg/ml vs. 0 pg/ml from ASC [*p*<0.001] and 18 ± 8 pg/ml from Kc [*p*<0.05]). This secretion from DF was drastically reduced in hyperglycemia (-72% at 25mM glucose, *p* = 0.001).

As noted, significantly higher KGF release was found for ASC and DF than for Kc in both glycemic conditions (*p*<0.001). KGF secretion was not affected by glycemia for each cell type.

**Fig 4:**
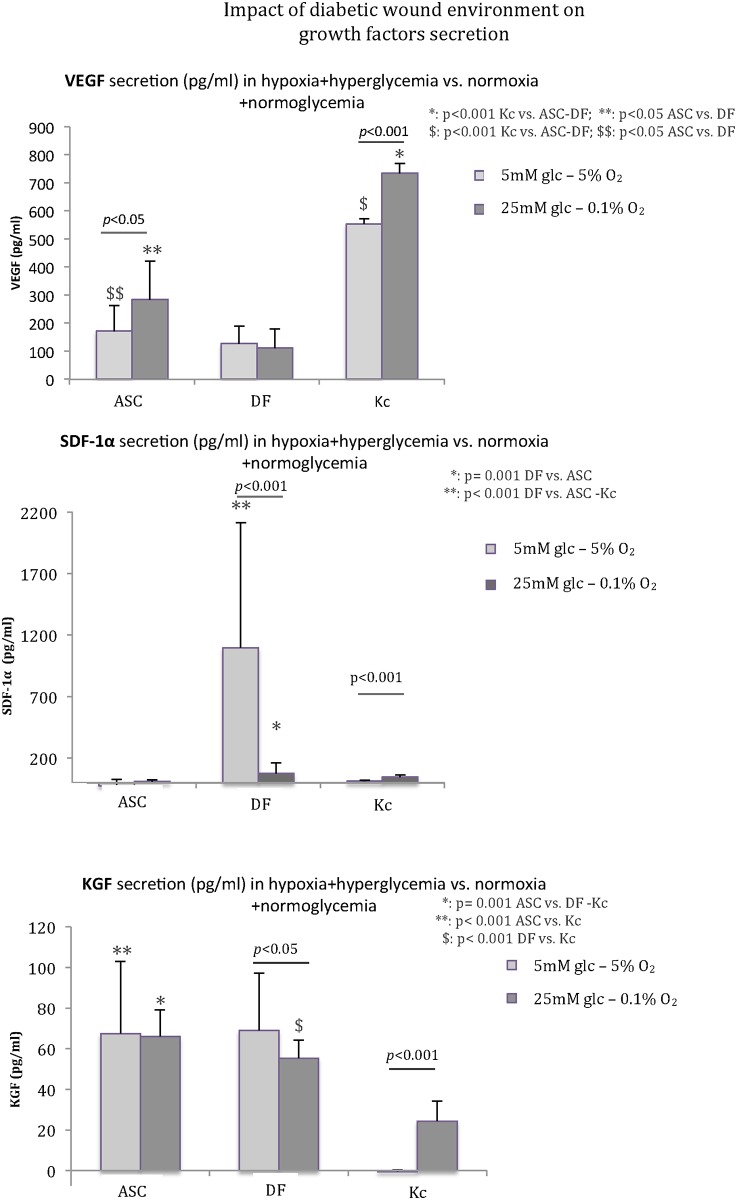
Impact of diabetic wound environment (hypoxia plus hyperglycemia) on VEGF, SDF-1α, and KGF secretion. In diabetic wound conditions, VEGF secretion by ASC was improved (*p*<0.05) but the hypoxic stimulus was lost for DF. Kc released a higher amount of VEGF and remained upregulated in hypoxia (*p*<0.001). SDF-1α secretion by DF was dramatically reduced in diabetic wound conditions (*p*<0.001). KGF secretion from ASC was not impacted in hypoxia or hyperglycemia, but DF secretion was altered (*p*<0.05). Kc released a higher quantity of KGF in the diabetic wound environment than in the physiological environment (*p*<0.001).

Finally, VEGF, SDF-1α, and KGF secretions were studied under conditions mimicking the diabetic wound environment (hypoxia plus hyperglycemia) compared to physiological conditions (tissular normoxia plus normoglycemia) (Fig 4). VEGF secretion was significantly increased in diabetic versus physiological conditions for ASC (*p*<0.05) and Kc (*p*<0.001). No modification was found for DF. VEGF secretion was higher for ASC (284 ± 134 pg/ml) than for DF (112 ± 68 pg/ml) in diabetic wound conditions (+253%, *p*<0.05) and physiological conditions (+35%, *p*<0.05). Hypoxia stimulus was lost in DF cultured in hyperglycemic media (25mM glucose), whereas ASC maintained their upregulation at 0.1% O_2_.

SDF-1α secretion by DF decreased in hypoxia plus hyperglycemia (75 ± 83 pg/ml vs. 1098 ±1017 pg/ml in physiological conditions, -93%, *p*<0.001). Conversely, Kc secretion was higher in hypoxia plus hyperglycemia (47 ± 15 pg/ml vs. 14 ± 8 pg/ml in physiological conditions, *p*<0.001).

KGF secretion was maintained for ASC (66 ± 13 pg/ml in diabetic vs. 67 ± 36 pg/ml in physiological conditions) and altered for DF in hypoxia plus hyperglycemia (-20%: 55 ± 9 pg/ml vs. 69 ± 28 pg/ml in physiological conditions, *p*<0.05). Regarding Kc, secretion was significantly higher in diabetic wound conditions (24 ± 10 pg/ml vs. no secretion in physiological conditions, *p*<0.001) but remained lower than the release from ASC (*p* = 0.001) and DF (*p*<0.001). Significantly higher KGF secretion was observed from ASC when compared to DF (*p* = 0.001) and Kc (*p* = 0.001) in hypoxia plus hyperglycemia.

**Fig 5:**
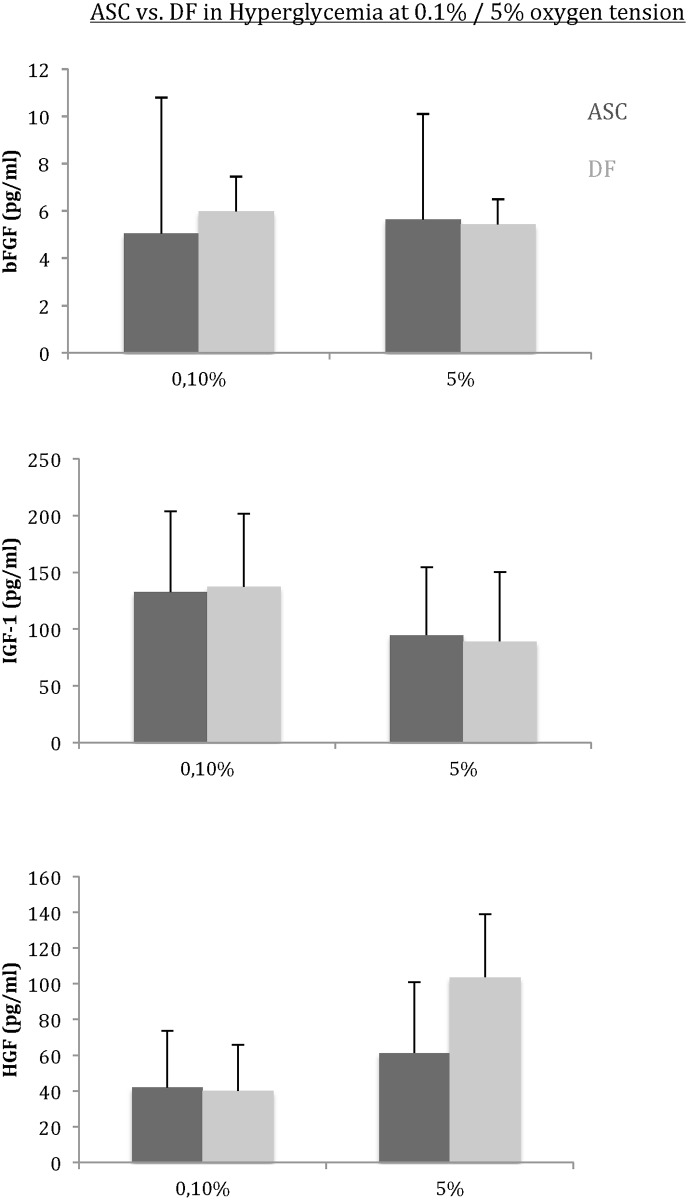
Impact of hypoxia plus hyperglycemia on IGF-1, HGF and bFGF secretion. No significant variation of growth factors secretion was found between DF and ASC in each condition. In addition, the hypoxia and hyperglycemia did not affect the secretion of these growth factors by DF and ASC, respectively.

No impact of glucose concentration and oxygen level was found on the secretion of IGF-1, bFGF and HGF for ASC and DF (Fig 5).

### II: Impact of oxygen tension and glucose concentration on ASC from type 2 diabetic patients

**Fig 6:**
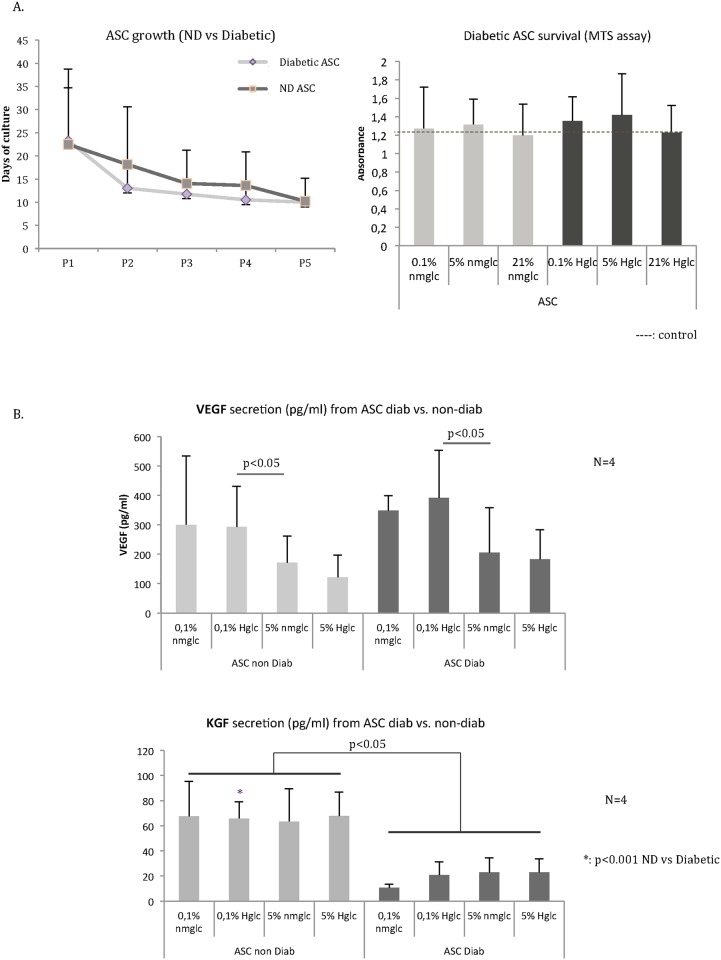
**A. Growth of ASC from type 2 diabetes patients (n = 4) was compared to the growth of ASC from nondiabetic patients (ND ASC, n = 8)**. No significant difference was observed between the two populations. The mean expansion time to passage 4 was 58.5 days (±18 days) for diabetic ASC versus 62.5 days (±28 days) for ND ASC (*p* = NS). Survival in standard culture conditions was considered control (*). Diabetic ASC survival (right) was not significantly impacted by oxygen level (0.1%, 5%, or 21% O_2_) or by glycemia in the culture media (5 mM or 25 mM glucose). **B. Comparison of VEGF secretion in type 2 diabetes ASC and in ND ASC** revealed a significant increase in chronic wound conditions that was maintained in both groups (diabetic and nondiabetic ASC, *p*<0.05) in comparison to physiological conditions (5% O_2_ tension and 5 mM glucose). Regarding KGF secretion, a significant decrease was found for diabetic ASC in diabetic wound conditions (*p*<0.001) and physiological conditions (*p*<0.05) in comparison to nondiabetic ASC.

ASC harvested from type 2 diabetes patients (n = 4) were isolated and expanded until passage 4 or 5 in standard culture conditions (Table 3). The growth rate of diabetic ASC was similar to that of nondiabetic ASC (ND ASC). The mean expansion time up to passage 4 was 58 (±18) days for diabetic ASC versus 62 (±28) days for ND ASC (*p* = NS). Cell survival was not modified after incubation at 0.1%, 5%, or 21% O_2_ and 5 mM or 25 mM glucose (Fig 6A).

**Table 3:** Type 2 diabetic ASC donor characteristics.

Donor	1	2	3	4
**Age (years)**	53	71	56	55
**Sex**	M	M	F	F
**Anatomical site**	abdomen	Abdomen	Breast	abdomen
**HbA1c**	8.1%	5.1%	7.3%	7%
**MD2 duration (years)**	10	15	11	5
**Treatment**	Biguanide	Sulfonylurea	Sulfonylurea	Biguanide

VEGF and KGF secretions from diabetic ASC were compared to secretions from ND ASC (Fig 6B). No significant differences were observed between ND and diabetic ASC for VEGF secretions in each condition. A significantly higher release of VEGF was found at 0.1% O_2_ and 25mM glucose for both ND and diabetic ASC in comparison to physiological conditions (294 ± 119 pg/ml vs. 172 ± 90 pg/ml for ND ASC and 392 ± 145 pg/ml vs. 206 ± 139 pg/ml for diabetic ASC, respectively, *p*<0.05). However, KGF secretion was significantly reduced for diabetic ASC in physiological conditions (23 ± 9 pg/ml, corresponding to 34% of ND ASC secretion, *p*<0.05) as well as in hypoxia plus hyperglycemia (21 ± 11 pg/ml, corresponding to 31.7% of ND ASC secretion, *p*<0.001).

## Discussion

Regarding ASC use for diabetic wounds, we first (before *in vivo* preclinical and clinical studies) need to demonstrate the capacity to isolate ASC from adipose tissue of diabetic patients, the capacity of to survive in a diabetic environment for cellular engraftment, and the ability of ASC to secrete the appropriate growth factor profile at very low oxygen levels and during hyperglycemia to promote angiogenesis and tissue remodeling. ASC were compared *in vitro* to Kc and DF already used clinically in biological skin equivalents (such as Apligraf^®^ and Dermagraft^®^, respectively) [32,33, 34].

Although we previously demonstrated the capacity to reproducibly isolate a pure population of human ASC for the manufacturing of a biological bandage for skin reconstruction [28, 35, 36], the future clinical success of ASC for diabetic wounds is based on the assumption that the cell population (obtained from the adipose tissue of diabetic patients) is equivalent to that obtained from non-diabetic patients. Using Good Manufacturing Practice, heterogeneity introduced by “macro-differences” (starting material, isolating methods, cell production processes) and “micro-differences” (donor-to-donor variability, donor conditions at the time of sampling) could also significantly modify the cell manufacturing processes and the quality of the final cell therapy product. In the present study, we investigated the isolation of ASC from diabetic patients and the subsequent ability of these cells to survive and proliferate in stress conditions such as hypoxia and hyperglycemia. Our data suggest that diabetes does not significantly affect ASC isolation efficiency and proliferation, as found by Policha et al. [37]. Cellular senescence, found in diabetic adipose tissue, did not influence ASC isolation because passage 1 was obtained during the exact same range of time as non-diabetic ASC [20,21]. A similar cellular growth curve was also found between non-diabetic and diabetic ASC up to passages 4 and 5, which corresponds to the cellular population required to promote (in our experience) VEGF release *in vitro* and angiogenesis for dermis regeneration *in vivo* [28,35,36]. We also found that hypoxia coupled with hyperglycemia did not affect ASC survival. In addition to this later finding, ASC (from healthy and diabetic patients) had better resistance to hypoxia and hyperglycemia than DF and Kc due to a lower proliferation rate, indicating a lower cellular turnover. Our results confirmed the fact that fibroblasts and Kc are specifically affected by hyperglycemia with senescence and decreased proliferation and that Kc also exhibit impaired migration [34]. Although diabetic status does not influence the source of ASC for cellular isolation and expansion, the stem cell function could be impaired in the hostile environment of a diabetic wound (severe hypoxia/hyperglycemia) [10,21,32–34].

Subsequently, this study compared the secretion of VEGF, SDF-1α (for their angiogenic properties) and KGF (promoting migration and proliferation of Kc) by diabetic vs. nondiabetic ASC [2,38–40]. A similar pattern of VEGF secretion was found for diabetic and nondiabetic ASC in all *in vitro* conditions with stimulation of VEGF release in hypoxia and hyperglycemia. In contrast, secretion of KGF was significantly depleted in hypoxia and hyperglycemia for ASC from type 2 diabetes patients in comparison to nondiabetic ASC. Although the hypoxia alone stimulated the release of VEGF by ASC, Kc, and DF, a significant stimulation of this latest secretion was found when ASC were exposed to hypoxia simultaneously coupled to hyperglycemia. In contrast, the secretion of VEGF was not improved when for DF was exposed to hyperglycemia and hypoxia. Interestingly, secretion of SDF-1α (only produced by DF in physiological conditions) decreased to the levels of ASC and Kc release in diabetic (hypoxia + hyperglycemia) conditions. A dramatic decrease in KGF release was also found for DF in hypoxia and hyperglycemia when ASC maintained their high level of secretion.

Recently, ASC have been found to secrete exosomes, which were recently described as a major effector in wound healing. After local or systemic injection (intra-veinously), a significant faster wound healing was found in a mouse model than untreated recipients [41]. The exosomes (small membrane vesicles derived from cellular compartments that fuses with the plasma membrane) are now considered as vital mediators of cellular communication and tissue remodelling for regenerative medicine by transferring membrane proteins, mRNAs, and miRNAs to recipient cells [42–44]. The exosomes can control the inflammatory response as found in the severe burn-induced excessive inflammation [45] and promote (with exosomes obtained from human adipose stem cells in a conditioned medium) in vitro and in vivo the skin dermal fibroblasts migration, proliferation and collagen synthesis [46]. Although Patel et al. demonstrated no impact of lean and obese donors in term of ASCs—exosomes secretion (for end-used in regenerative medicine) and a higher cellular senescence for ASCs from non-obese patients [47], a lowest KGF secretion was found in our hand for diabetic ASC with a similar cellular growth between ASC from non- and diabetic human donors. This difference between Patel et al. and our results could be explained by the fact that obese donors were not reported as diabetic under hypoglycemic drugs in the Patel’s study. Therefore, in view to assess the potential of autologous ASC for diabetic wound healing, the profile of the secretome of diabetic-ASC (with the cellular interaction for wound remodelling) need to be investigate before in vivo preclinical studies in a relevant animal model.

## Conclusions

Since the impact of chronic diabetes on ASC properties was still not well understood, this study demonstrated that human ASC can be proposed to cure diabetic wounds because of: (i) a similar capacity of ASC isolation and expansion in diabetic patients (than healthy patients), (ii) the capacity to survive in hypoxia and hyperglycemia (in contrast to Kc) and (iii) the expression of a better growth factor secretion profile than Kc and DF in hypoxia and hyperglycemia. However, the reduction of KGF secretion by ASC harvested from diabetic patients need to be investigate in a relevant preclinical diabetic model. It is reasonable to conclude that diabetic adipose tissue is a promising source of ASC for skin tissue engineering in the diabetic population. It appears that adipose tissue may be the preferred source of cells (in comparison to fibroblasts and Kc) for investigating wound healing in a diabetic model.

## Abbreviations

ASC: adipose-derived stem cells
DF: dermal fibroblasts
EdU: 5-ethynyl-2’-deoxyuridine
FBS: fetal bovine serum
Kc: keratinocytes
KGF: keratinocyte growth factor
MTS: 3-(4,5-dimethylthiazol-2-yl)-5-(3-carboxymethoxyphenyl)-2-(4-sulfophenyl)-2H-tetrazolium solution
SDF-1α: stromal cell-derived factor-1α
VEGF: vascular endothelial growth factor

## References

[1] Boulton AJ, Vileikyte L, Ragnarson-Tennvall G, Apelqvist J. The global burden of diabetic foot disease. *Lancet*. 2005;366: 1719–1724. 10.1016/S0140-6736(05)67698-2 16291066

[2] Brem H, Tomic-Canic M. Cellular and molecular basis of wound healing in diabetes. *J Clin Invest*. 2007;117: 1219–1222. 10.1172/JCI32169 17476353 PMC1857239

[3] Lerman OZ, Galiano RD, Armour M, Levine JP, Gurtner GC. Cellular dysfunction in the diabetic fibroblast: impairment in migration, vascular endothelial growth factor production, and response to hypoxia. *Am J Pathol*. 2003;162: 303–312. 10.1016/S0002-9440(10)63821-7 12507913 PMC1851127

[4] Thangarajah H, Yao D, Chang EI, Shi Y, Jazayeri L, Vial IN et al The molecular basis for impaired hypoxia-induced VEGF expression in diabetic tissues. *Proc Natl Acad Sci USA*. 2009;106: 13505–13510. 10.1073/pnas.0906670106 19666581 PMC2726398

[5] Thangarajah H, Vial IN, Grognan RH, Yao D, Shi Y, Januszyk M et al HIF-1alpha dysfunction in diabetes. *Cell Cycle*. 2010;9: 75–79. 10.4161/cc.9.1.10371 20016290

[6] Rennert RC, Sorkin M, Januszyk M, Duscher D, Kosaraju R, Chung MT et al Diabetes impairs the angiogenic potential of adipose-derived stem cells by selectively depleting cellular subpopulations. *Stem Cell Res Ther*. 2014;5: 79 10.1186/scrt468 24943716 PMC4097831

[7] Ma C, Hernandez MA, Kirkpatrick VE, Liang LJ, Nouvong AL, Gordon II. Topical platelet-derived growth factor vs placebo therapy of diabetic foot ulcers offloaded with windowed casts: a randomized, controlled trial. *Wounds*. 2015;27: 83–89. 25855851

[8] Zuk PA, Zhu M, Ashjian P, De Ugarte DA, Huang JI, Mizuno H et al Human adipose tissue is a source of multipotent stem cells. *Mol Biol Cell*. 2002;13: 4279–4295. 10.1091/mbc.E02-02-0105 12475952 PMC138633

[9] Vériter S, Aouassar N, Adnet PY, Paridaens MS, Stuckman C, Jordan B et al The impact of hyperglycemia and the presence of encapsulated islets on oxygenation within a bioartificial pancreas in the presence of mesenchymal stem cells in a diabetic Wistar rat model. *Biomaterials*. 2011;32: 5945–5956. 10.1016/j.biomaterials.2011.02.061 21676459

[10] Nie C, Yang D, Xu J, Si Z, Jin X, Zhang J. Locally administered adipose-derived stem cells accelerate wound healing through differentiation and vasculogenesis. *Cell Transplant*. 2011;20: 205–216. 10.3727/096368910X520065 20719083

[11] Wu Y, Chen L, Scott PG, Tredget EE. Mesenchymal stem cells enhance wound healing through differentiation and angiogenesis. *Stem Cells*. 2007;25: 2648–2659. 10.1634/stemcells.2007-0226 17615264

[12] Amos PJ, Kapur SK, Stapor PC, Shang H, Bekiranov S, Khurgel M et al Human adipose-derived stromal cells accelerate diabetic wound healing: impact of cell formulation and delivery. *Tissue Eng Part A*. 2010;16: 1595–1606. 10.1089/ten.TEA.2009.0616 20038211 PMC2952117

[13] Di Rocco G, Gentile A, Antonini A, Ceradini F, Wu JC, Capogrossi MC, Toietta G. Enhanced healing of diabetic wounds by topical administration of adipose tissue-derived stromal cells overexpressing overexpressing stromal-derived factor-1: biodistribution and engraftment analysis by bioluminescent imaging. *Stem Cells Int*. 2010;2011: 304562 10.4061/2011/304562 21234108 PMC3014681

[14] Nambu M, Kishimoto S, Nakamura S, Mizuno H, Yanagibayashi S, Yamamoto N et al Accelerated wound healing in healing impaired db/db mice by autologous adipose tissue-derived stromal cells combined with atelocollagen matrix. *Ann Plast Surg*. 2009;62: 317–321. 10.1097/SAP.0b013e31817f01b6 19240532

[15] Kim EK, Li G, Lee TJ, Hong JP. The effect of human adipose-derived stem cells on healing of ischemic wounds in a diabetic nude mouse model. *Plast Reconstr Surg*. 2011;128: 387–394. 10.1097/PRS.0b013e31821e6de2 21788830

[16] El-Ftesi S, Chang EI, Longaker MT, Gurtner GC. Aging and diabetes impair the neovascular potential of adipose-derived stromal cells. *Plast Reconstr Surg*. 2009;123: 475–485. 10.1097/PRS.0b013e3181954d08 19182604 PMC2878769

[17] Yan J, Tie G, Wang S, Messina KE, DiDato S, Guo S, Messina LM. Type 2 diabetes restricts multipotency of mesenchymal stem cells and impairs their capacity to augment postischemic neovascularization in db/db mice. *J Am Heart Assoc*. 2012;1: e002238 10.1161/JAHA.112.002238 23316315 PMC3540677

[18] Cianfarani F, Toietta G, Di Rocco G, Cesareo E, Zambruno G, Odorisio T. Diabetes impairs adipose tissue-derived stem cell function and efficiency in promoting wound healing. *Wound Repair Regen*. 2013;21: 545–553. 10.1111/wrr.12051 23627689

[19] Desmet CM, Lafosse A, Vériter S, Porporato PE, Sonveaux P, Dufrane D et al Application of electron paramagnetic resonance (EPR) oximetry to monitor oxygen in wounds in diabetic models. *PLoS One*. 2015;10: e0144914 10.1371/journal.pone.0144914 26659378 PMC4679295

[20] Minamino T, Orimo M, Shimizu I, Kunieda T, Yokoyama M, Ito T et al A crucial role for adipose tissue p53 in the regulation of insulin resistance. *Nat Med*. 2009;15: 1082–1087. 10.1038/nm.2014 19718037

[21] Shimizu I, Yoshida Y, Suda M, Minamino T. DNA damage response and metabolic disease. *Cell Metab*. 2014;20: 967–977. 10.1016/j.cmet.2014.10.008 25456739

[22] Johnson KE, Wilgus TA. Vascular Endothelial Growth Factor and Angiogenesis in the Regulation of Cutaneous Wound Repair. *Adv Wound Care*. 2014;3: 647–661. 10.1089/wound.2013.0517 PMC4183920 25302139

[23] Xu X, Zhu F, Zhang M, Zeng D, Luo D, Liu G et al Stromal cell-derived factor-1 enhances wound healing through recruiting bone marrow-derived mesenchymal stem cells to the wound area and promoting neovascularization. *Cells Tissues Organs*. 2013;197: 103–113. 10.1159/000342921 23207453

[24] Gallagher KA, Liu ZJ, Xiao M, Chen H, Goldstein LJ, Buerk DG et al Diabetic impairments in NO-mediated endothelial progenitor cell mobilization and homing are reversed by hyperoxia and SDF-1 alpha. *J Clin Invest*. 2007;117: 1249–1259. 10.1172/JCI29710 17476357 PMC1857264

[25] Marti GP, Mohebi P, Liu L, Wang J, Miyashita T, Harmon JW. KGF-1 for wound healing in animal models. *Methods in Molecular Biology*. 2008;423:383–391. 10.1007/978-1-59745-194-9_30 18370216

[26] Seeger MA, Paller AS. The Roles of Growth Factors in Keratinocyte Migration. *Adv Wound Care (New Rochelle)*. 2015;4: 213–224. 25945284 10.1089/wound.2014.0540 PMC4397993

[27] Coleman SR. Structural fat grafts: the ideal filler? *Clin Plast Surg*. 2001;28: 111–119. 11248861

[28] Lafosse A, Desmet C, Aouassar N, André W, Hanet MS, Beauloye C et al Autologous adipose stromal cells seeded onto a human collagen matrix for dermal regeneration in chronic wounds: Clinical proof of concept. *Plast Reconstr Surg*. 2015;136: 279–295. 10.1097/PRS.0000000000001437 25946602

[29] Qu CQ, Zhang GH, Zhang LJ, Yang GS. Osteogenic and adipogenic potential of porcine adipose mesenchymal stem cells. *In Vitro Cell Dev Biol Anim*. 2007;43: 95–100. 10.1007/s11626-006-9008-y 17570023

[30] Schubert T, Lafont S, Beaurin G, Grisay G, Behets C, Gianello P, Dufrane D. Critical size bone defect reconstruction by an autologous 3D osteogenic-like tissue derived from differentiated adipose MSCs. *Biomaterials*. 2013;34: 4428–4438. 10.1016/j.biomaterials.2013.02.053 23507085

[31] De Meester C, Timmermans AD, Balteau M, Ginion A, Roelants V, Noppe G et al Role of AMP-activated protein kinase in regulating hypoxic survival and proliferation of mesenchymal stem cells. *Cardiovasc Res*. 2014; 101:20–29. 10.1093/cvr/cvt227 24104879

[32] Harding K, Sumner M, Cardinal M. A prospective, multicentre, randomised controlled study of human fibroblast-derived dermal substitute (Dermagraft) in patients with venous leg ulcers. *Int Wound J*. 2013; 10:132–137. 10.1111/iwj.12053 23506344 PMC7950758

[33] Zelen CM, Gould L, Serena TE, Carter MJ, Keller J, Li WW. A prospective, randomised, controlled, multi-centre comparative effectiveness study of healing using dehydrated human amnion/chorion membrane allograft, bioengineered skin substitute or standard of care for treatment of chronic lower extremity diabetic ulcers. *Int Wound J*. 2015; 12:724–732. 10.1111/iwj.12395 25424146 PMC7950807

[34] Lev-Tov H, Li CS, Dahle S, Isseroff RR. Cellular versus acellular matrix devices in treatment of diabetic foot ulcers: study protocol for a comparative efficacy randomized controlled trial. *Trials*. 2013; 14:8 10.1186/1745-6215-14-8 23298410 PMC3553036

[35] Dufrane D and Lafosse A. A simple method to determine the purity of adipose-derived stem cell-based cell therapies. *Stem Cells Transl Med*. 2016. 10.5966/sctm.2016-0013 PMC5070509 27400794

[36] Vériter S, André W, Aouassar N, Poirel HA, Lafosse A, Docquier PL et al Human adipose-derived mesenchymal stem cells in cell therapy: safety and feasibility in different "hospital exemption" clinical applications. *PLoS One*. 2015;10: e0139566 10.1371/journal.pone.0139566 26485394 PMC4615620

[37] Policha A, Zhang P, Chang L, Lamb K, Tulenko T, DiMuzio P. Endothelial differentiation of diabetic adipose-derived stem cells. *J Surg Res*. 2014;192: 656–663. 10.1016/j.jss.2014.06.041 25091340

[38] Kapur SK, Katz AJ. Review of the adipose derived stem cell secretome. *Biochimie*. 2013;95: 2222–2228. 10.1016/j.biochi.2013.06.001 23770442

[39] Johnson KE, Wilgus TA. Vascular endothelial growth factor and angiogenesis in the regulation of cutaneous wound repair. *Adv Wound Care*. 2014;3: 647–661. 10.1089/wound.2013.0517 PMC4183920 25302139

[40] Xu X, Zhu F, Zhang M, Zeng D, Luo D, Liu G et al Stromal cell-derived factor-1 enhances wound healing through recruiting bone marrow-derived mesenchymal stem cells to the wound area and promoting neovascularization. *Cells Tissues Organs*. 2013;197: 103–113. 10.1159/000342921 23207453

[41] Hu L, Wang J, Zhou X, Xiong Z, Zhao J, Yu R et al Exosomes derived from human adipose mensenchymal stem cells accelerates cutaneous wound healing via optimizing the characteristics of fibroblasts.*Sci Rep*. 2016:6:32993 10.1038/srep32993 27615560 PMC5018733

[42] Hoshino D, Kirkbride KC, Costello K, Clark ES, Sinha S, Grega-Larson N et al, Tyska M. Exosome secretion is enhanced by invadopodia and drives invasive behavior. *Cell Rep*. 2013;5:1159–1168. 10.1016/j.celrep.2013.10.050 24290760 PMC3873329

[43] Hu G, Drescher KM, Chen XM. Exosomal miRNAs: biological properties and therapeutic potential. *Front*. *Genet*. 2012;3:56 10.3389/fgene.2012.00056 22529849 PMC3330238

[44] Kowal J, Tkach M, Thery C. Biogenesis and secretion of exosomes. *Curr*. *Opin*. *Cell Biol*. 2014;29:116–125. 10.1016/j.ceb.2014.05.004 24959705

[45] Li X, Liu L, Yang J, Yu Y, Chai J, Wang L, Ma L, Yin H. Exosome Derived From Human Umbilical Cord Mesenchymal Stem Cell Mediates MiR-181c Attenuating Burn-induced Excessive Inflammation. *EBioMedicine*. 2016;8:72–82. 10.1016/j.ebiom.2016.04.030 27428420 PMC4919539

[46] Hu L, Wang J, Zhou X, Xiong Z, Zhao J, Yu R, et al Exosomes derived from human adipose mensenchymal stem cells accelerates cutaneous wound healing via optimizing the characteristics of fibroblasts. *Sci Rep*. 2016;6:32993 10.1038/srep32993 27615560 PMC5018733

[47] Patel RS, Carter G, El Bassit G, Patel AA, Cooper DR, Murr M et al Adipose-derived stem cells from lean and obese humans show depot specific differences in their stem cell markers, exosome contents and senescence: role of protein kinase C delta (PKCδ) in adipose stem cell niche. *Stem Cell Investig*. 2016;3:2 10.3978/j.issn.2306-9759.2016.01.02 27358894 PMC4923648

